# Supplementary material for: Benzalkonium Chloride and Benzethonium Chloride Effectively Reduce Spore Germination of Ginger Soft Rot Pathogens: Fusarium solani and Fusarium oxysporum
Source: J Fungi (Basel). 2023 Dec 22;10(1):8. doi: 10.3390/jof10010008 (PMC10816859; doi:10.3390/jof10010008)
Supplement: Supplementary file 1 [file jof-10-00008-s001.zip › jof-2770193-supplementary.pdf]

Supporting Information

Benzalkonium Chloride and Benzethonium Chloride Effectively Reduce Spore Germination of Ginger Soft Rot Pathogens: *Fusarium solani* and *Fusarium oxysporum*

Dongxu Zhao [1], Yang Zhang [1], Zhaoyang Jin [1], Ruxiao Bai [2], Jun Wang [2], Li Wu [1,3,*] and Yujian He [1,2,4,*]

[1] School of Chemical Sciences, University of Chinese Academy of Sciences, Beijing 100049, China
[2] Institute of Farmland Water Conservancy and Soil Fertilizers, Xinjiang Academy of Agricultural Reclamation Sciences, Shihezi 832000, China
[3] State Key Laboratory of Natural and Biomimetic Drugs, School of Pharmaceutical Sciences, Peking University, Beijing 100191, China
[4] School of Future Technology, University of Chinese Academy of Sciences, Beijing 100049, China
* Correspondence: wuli@ucas.ac.cn (L.W.); heyujian@ucas.ac.cn (Y.H.)

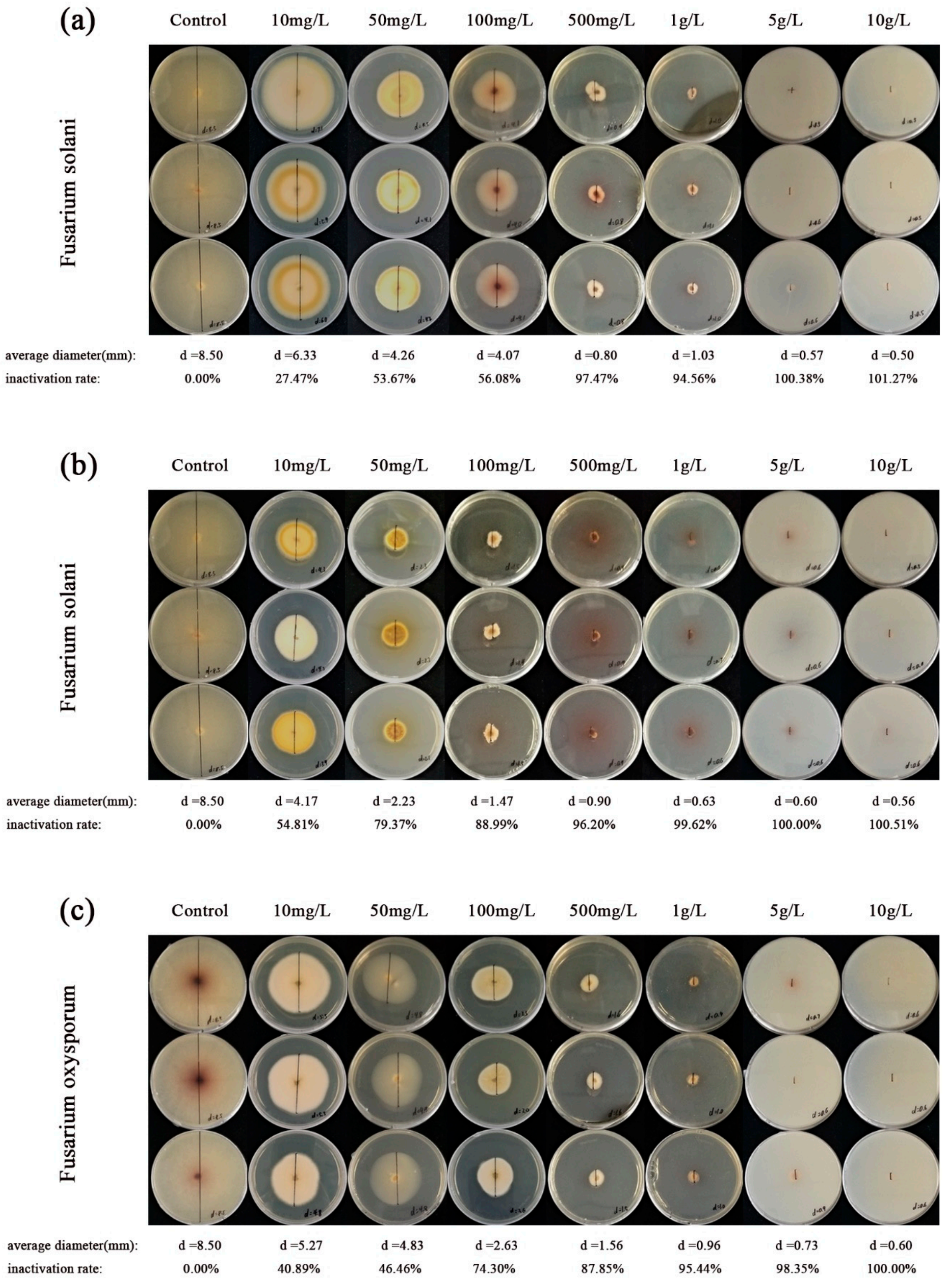
(a)
Control
10mg/L
50mg/L
100mg/L
500mg/L
1g/L
5g/L
10g/L
Fusarium solani
average diameter(mm): d =8.50 d =6.33 d =4.26 d =4.07 d =0.80 d =1.03 d =0.57 d =0.50
inactivation rate: 0.00% 27.47% 53.67% 56.08% 97.47% 94.56% 100.38% 101.27%
(b)
Control
10mg/L
50mg/L
100mg/L
500mg/L
1g/L
5g/L
10g/L
Fusarium solani
average diameter(mm): d =8.50 d =4.17 d =2.23 d =1.47 d =0.90 d =0.63 d =0.60 d =0.56
inactivation rate: 0.00% 54.81% 79.37% 88.99% 96.20% 99.62% 100.00% 100.51%
(c)
Control
10mg/L
50mg/L
100mg/L
500mg/L
1g/L
5g/L
10g/L
Fusarium oxysporum
average diameter(mm): d =8.50 d =5.27 d =4.83 d =2.63 d =1.56 d =0.96 d =0.73 d =0.60
inactivation rate: 0.00% 40.89% 46.46% 74.30% 87.85% 95.44% 98.35% 100.00%

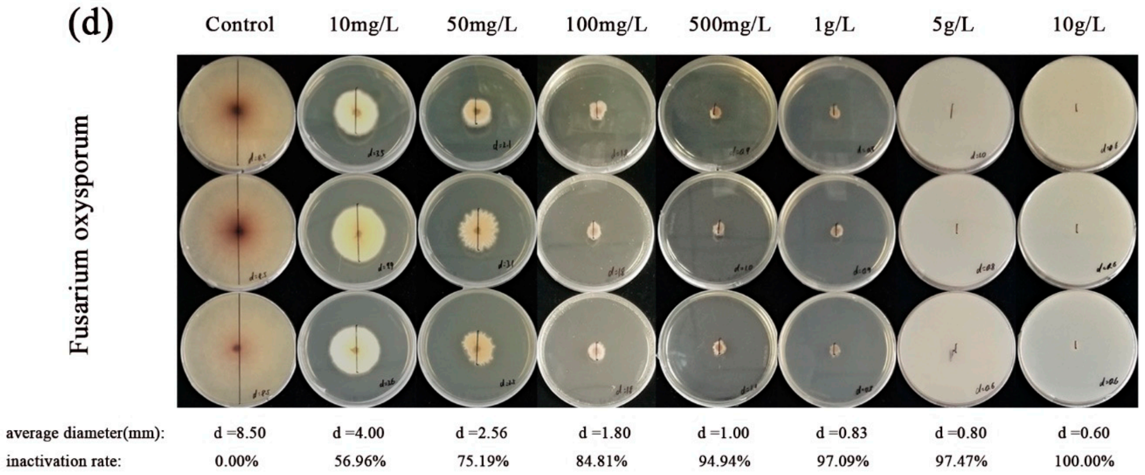

Figure S1. Comparison of mycelial growth of the two fungi after 7 days of incubation on fungicide-containing plates (Corresponds to the bar chart in Figure 4). (a) *Fusarium solani* mycelium after BAC treatment; (b) *Fusarium solani* after BEC treatment; (c) *Fusarium oxysporum* mycelium after BAC treatment; (d) *Fusarium oxysporum* mycelium after BEC treatment. Experimental conditions: [BAC] = [BEC] = 10, 50, 100, 500, 1000, 5000 mg/L; initial concentration of fungal spores = 10^6 CFU/mL; pH = 7.40; T = 28±1 °C.

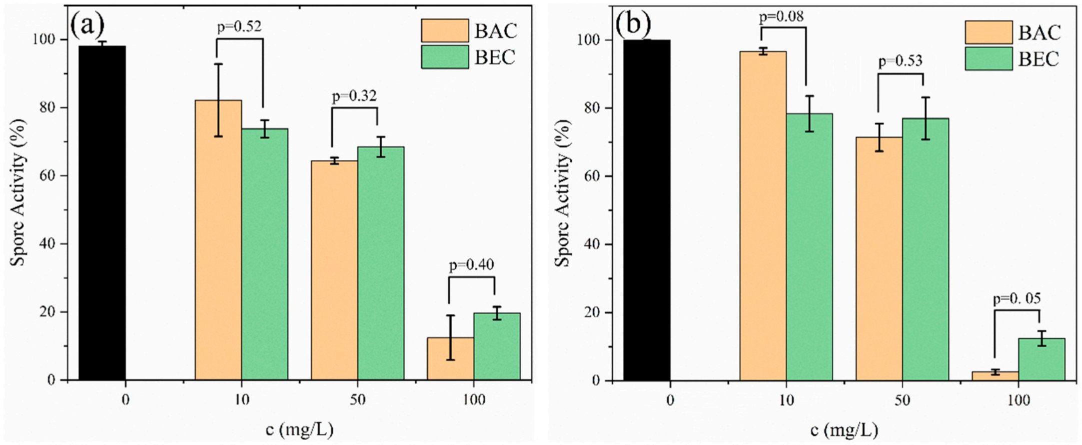

Figure S2. Laser confocal microscopy to observe changes in spore viability. Expressed as the ratio of red fluorescence intensity to total fluorescence intensity. (a) *Fusarium solani* spores and (b) *Fusarium oxysporum* spores. Experimental conditions: initial concentration of fungal spores = 10^6 CFU/mL; pH = 7.40; T = 28±1 °C.

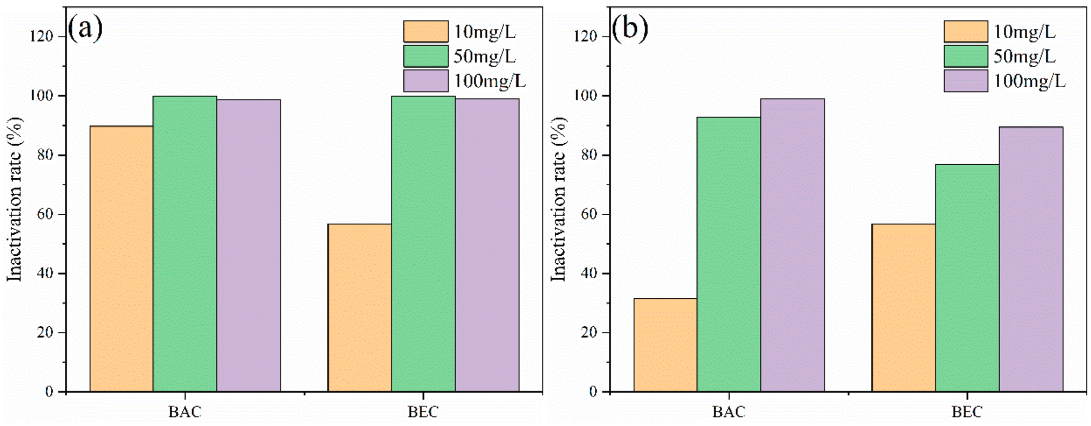

Figure S3. Results of flow cytometric analysis of inactivation of *Fusarium solani* spores (a) and *Fusarium oxysporum* spores (b) by BAC and BEC. Experimental conditions: [BAC] = [BEC] = 10, 50, 100 mg/L; initial concentration of fungal spores = 10^6 CFU/mL; pH = 7.40; T = 28±1 °C.

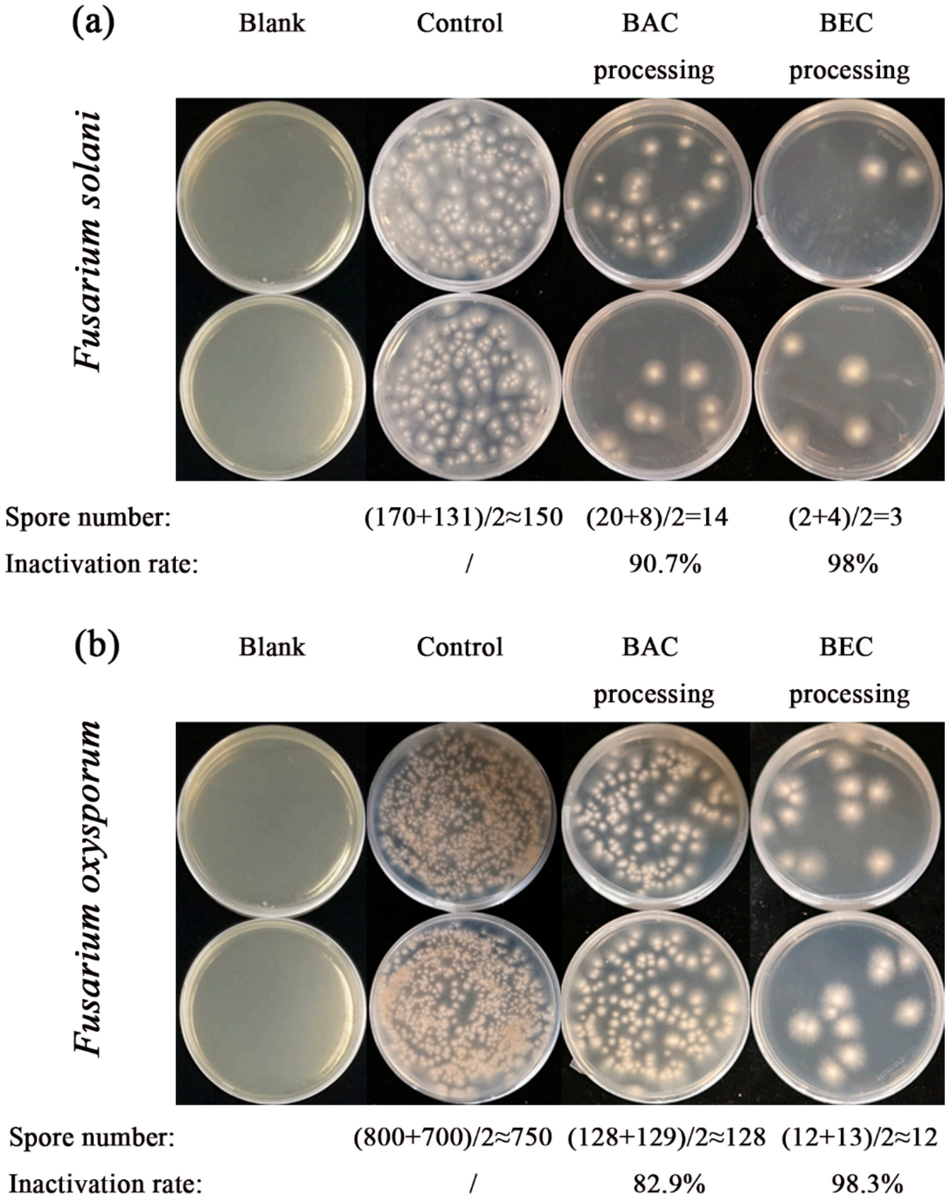

Figure S4. Effect of BAC and BEC on the inactivation of *Fusarium solani* spores (a) and *Fusarium oxysporum* spores (b) in sterilized soil samples. Experimental conditions: [BAC] = [BEC] = 100mg/L; initial concentration of fungal spores = 10^6 CFU/mL; pH = 7.40; T = 28±1 °C; reaction time= 30 min.
